# Characteristics and influencing factors of gut microbiota in population with sleep disorders

**DOI:** 10.3389/fmicb.2025.1586864

**Published:** 2025-07-10

**Authors:** Chunhong Zhang, Qinglin Sheng, Yuanqi Wang, Qi Shen, Yifei Zhai, Dawei Hu, Nanjia Zhang, Ziyuan Wang, Xuebin Yin, Dan Li, Youtao Chen

**Affiliations:** ^1^Institute of Food Science and Technology, CAAS, Beijing, China; ^2^Guangdong Provincial Key Laboratory of Intelligent Port Security Inspection, Guangzhou, China; ^3^Anhui Province Key Laboratory of Functional Agriculture and Functional Food, Anhui Science and Technology University, Chuzhou, China; ^4^School of Health Science and Engineering, University of Shanghai for Science and Technology, Shanghai, China; ^5^School of Basic Medical Science, Naval Medical University, Shanghai, China; ^6^College of Agricultural Engineering and Food Science, Shandong University of Technology, Zibo, China; ^7^PLA Navy Medical Center, Shanghai, China

**Keywords:** insomnia, gut, oral cavity, microbial flora, metabolomics

## Abstract

**Introduction:**

The integrated analysis of gut and oral microbiota and their metabolites helps elucidate key factors affecting sleep disorders in populations and provides research insights for understanding sleep regulation mechanisms.

**Methods:**

Based on a cross-sectional study design, this research combined 16S sequencing and untargeted metabolomics to investigate lifestyle habits and physical conditions of 165 adult male subjects, systematically examining characteristics of gut and oral microbiota and their metabolites.

**Results:**

Analysis of gut microbiota revealed significantly reduced microbial diversity in the insomnia group, with predominant phyla being *Firmicutes*, *Actinobacteriota*, and *Bacteroidetes*. At the genus level, the abundance of Blautia was significantly elevated. Gut metabolite analysis showed significant enrichment in metabolic pathways such as “phenylalanine, tyrosine, and tryptophan biosynthesis.” Regarding oral microbiota, no significant difference in diversity was observed between sleepless and normal groups. At the genus level, the sleepless group showed significantly decreased abundance of *Streptococcus* and increased abundance of *Veillonella*. Metabolite analysis indicated significant correlation between the sleepless group and metabolic pathways such as “pantothenate and CoA biosynthesis.”

**Discussion:**

This study compared differences in gut and oral microbiota and metabolites between sleepless and normal groups, identifying potential biomarkers for insomnia, including gut *Blautia*, aromatic amino acid metabolites, salivary *Streptococcus* and *Veillonella*, and pantothenate-related metabolites. These findings provide important multi-omics data for investigating the pathological mechanisms of insomnia. We have made changes according to the requirements, please adjust according to the standard.

## 1 Introduction

Statistically, 10–15% of adults worldwide suffer from chronic insomnia, and another 25–35% experience transient or occasional insomnia (Zhou et al., 2022). Sleep deprivation can have various adverse effects on human health, including immune-related diseases and metabolic disorders, such as rheumatoid arthritis, systemic lupus erythematosus, and diabetes (Weir and Price, 2019; Yang et al., 2022; Sun et al., 2023). Good sleep can regulate hormonal levels in the body, such as growth hormone and leptin, which are crucial for cardiovascular health and blood glucose metabolism (Fletcher and Frith, 2009). Deep sleep facilitates the conversion of short-term memory into long-term memory, and the brain’s “clearance system” during sleep can eliminate metabolic waste, reducing the risk of neurodegenerative diseases, promoting the reconnection of neural synapses, and enhancing the brain’s learning and adaptive abilities (Rasch and Born, 2013; Xie et al., 2013).

There is a bidirectional relationship between sleep quality and the gut microbiome. The gut microbiota participates in metabolic processes and acts as a regulatory factor (Wang et al., 2017). The gut microbiota not only affects the host’s digestion, metabolism, and immune function but also regulates the host’s sleep and psychological state through the microbiome-gut-brain axis (Ridaura et al., 2013; Seong et al., 2024). Neurotransmitters and metabolites produced by the gut microbiota can influence the neurons of the enteric nervous system, thereby affecting the neural circuits involved in sleep-wake regulation (Ojeda et al., 2021). For instance, indole compounds generated from tryptophan metabolism can act on the brain through the bloodstream, regulating the levels of serotonin (5-HT), thus significantly influencing mood, sleep patterns, and arousal cycles. Bile acid metabolites, on the other hand, affect neural circuit activity through inter-actions with enteroendocrine cells and the vagus nerve system. These metabolites not only function as signaling molecules regulating the host’s sleep-wake cycles but may also optimize sleep quality and neural health through anti-inflammatory and neuroprotective actions.

It is widely known that a healthy oral microbiome can prevent the host from being infected by opportunistic pathogens and contribute to maintaining oral health. The oral microbiome plays a crucial role in participating in the digestion process and assisting in nutrient absorption. However, it is noteworthy that relevant studies have also indicated that the oral microbiome has a certain impact on some intestinal diseases and diabetes. Bianchi et al. (2023) suggested that specific compositions of the oral microbiome may play a role in obstructive sleep apnea (OSA), a chronic respiratory disorder related to sleep. Nevertheless, there are limited studies directly linking the oral microbiome, its metabolic characteristics, and sleep, making it a worthwhile area for further investigation.

This study employs 16S sequencing and untargeted metabolomics techniques to analyze the gut and oral microbiome compositions, as well as their metabolite profiles, in individuals with insufficient sleep and normal sleep. The aim is to uncover potential associations between sleep, gut-oral digestive tract microbiomes, and metabolites, providing a reference for intervention targets to alleviate sleep disturbances in individuals with sleep disorders.

## 2 Materials and methods

### 2.1 Study participants

The study participants were adult males aged 18–60 years old. The exclusion criteria for participants were (1) individuals with diarrhea, diabetes, ulcerative colitis, Crohn’s disease, or other infectious diseases (except for the disease under study), (2) those who had undergone chemotherapy, radiotherapy, or surgery, (3) those who had taken antibiotics, corticosteroids, Chinese herbal medicines (oral, intramuscular, or intravenous), or probiotics (such as yogurt) within 3–6 months prior to sampling, (4) those with significant dietary changes within 1 week before sampling. This study was approved by the Ethics Committee of the Naval Medical Center of the Naval Medical University, with Approval Number AF-HEC-017. Prior to participation, participants were informed about the experimental procedures and potential risks, and signed an informed consent form.

### 2.2 Questionnaire survey

A cross-sectional survey method was employed to collect information about residents’ dietary habits and physical conditions through a questionnaire. The researchers designed the “Residents’ Dietary Habits and Physical Condition Questionnaire” based on literature review and practical considerations. This self-administered questionnaire consisted of three main sections: basic information, a brief questionnaire, and detailed information. The basic information section included gender, age, height, weight, and other details. The brief questionnaire covered dietary preferences, daily staple food proportions, daily food combinations, sleep patterns, bowel movement frequency, and other factors. The detailed information section encompassed dietary habits (such as commonly consumed staple foods, vegetables, and cooking methods), other habits (such as smoking, physical activity and exercise, sleep duration), and health status (such as diagnosed diseases, medication use, and dietary supplement consumption).

### 2.3 Sample collection and storage

On the evening before sample collection, participants were provided with sample containers. The next morning, participants collected saliva samples before brushing their teeth and collected 3–5 g of fecal samples within the past 3 days. The collected saliva and fecal samples were transported to a freezer room at the sampling site within a day and then delivered to the laboratory under cold chain conditions within 3 days, where they were stored at −80°C for further analysis.

### 2.4 Microbiome analysis

Total bacterial DNA was extracted from saliva samples using the E.Z.N.A.^®^ Soil DNA Kit, and from fecal samples using the QIAamp Fast Stool DNA Kit. DNA samples underwent 16S rRNA relative quantitative sequencing (conducted by Majorbio Company, Shanghai). Subsequent data analysis was performed using R software.

### 2.5 Metabolomics analysis

Equal volumes of samples were used to prepare quality control (QC) samples. During the analysis process, one QC sample was inserted for every 5–15 samples to monitor the stability of the entire analytical process. Samples were analyzed using the Thermo Fisher Scientific UHPLC-Q Exactive HF-X system for LC-MS/MS analysis.

### 2.6 Data analysis

Data analysis was performed using SPSS 27.0 software, and measurement data were presented as $\bar{X}\pm S$ . The *Χ*^2^ test was used to compare differences between groups, and a univariate logistic regression model was employed to analyze factors influencing participants’ emotional and psychological conditions, as well as sleep quality. A multivariate logistic regression model was used to adjust for potential confounding factors and analyze factors related to emotional and psychological conditions and sleep quality. All statistical tests were two-tailed probability tests, with a significance level of *a* = 0.05.

Alpha diversity was calculated using the Shannon index, and the Wilcoxon rank-sum test was used to evaluate differences between groups. Beta diversity was assessed using principal coordinate analysis (PCoA) at the OTU level to evaluate differences in community structure between samples. Partial least squares discriminant analysis (PLS-DA) was used to distinguish differences in gut microbiome composition between different groups, and LefSe analysis was used to identify significantly different taxa between groups based on LDA scores. Identified metabolites were annotated using the KEGG database. Principal component analysis (PCA) was used to investigate overall differences between samples. Orthogonal partial least squares discriminant analysis (OPLS-DA) was used to distinguish differences in gut microbiome metabolite composition between different groups. Volcano plots were used to identify significantly changed metabolites, with *p* < 0.05 considered statistically significant.

## 3 Results

### 3.1 Basic characteristics of study participants

This study included 165 participants, with 43 in the normal group and 122 in the sleepless group. The proportion of participants in the sleepless group was as high as 73.94%, reflecting the prevalence of sleep problems to some extent. Specifically, regarding daily sleep duration, the sleepless group had significantly lower sleep duration compared to the normal group (*p* < 0.05). All participants in the sleepless group slept less than 6 h per day (100%), while none in the normal group slept less than 6 h (*p* = 0.044), indicating that the sleepless group had significantly shorter sleep durations. In the sleep-less group, 85.7% of participants were often in a state of high tension, compared to 14.3% in the normal group, a significant difference (*p* = 0.01).

In the sleepless group, 69.2% of participants regularly consumed probiotic products, significantly higher than the 30.8% in the normal group (*p* = 0.042). Sleep quality was significantly different between the two groups (*p* < 0.01), with 90.8% of participants in the sleepless group reporting “general” sleep conditions, compared to 9.2% in the normal group. In the sleepless group, 79.2% of participants preferred acidic foods, while in the normal group, this proportion was only 20.8%, a significant difference (*p* = 0.012). In the sleepless group, 79.1% of participants had a staple food-based diet, while in the normal group, this proportion was 20.9%, a significant difference (*p* < 0.01) (see Table 1).

**Table 1:** Analysis of sleep-related factors.

Items		Normal group (*N* = 43)	Sleepless group (*N* = 122)	*Χ*^2^ value	*p*
Daily sleep duration	<6 h	0a (0.0)	16a (100.0)	4.17	0.044
	6–8 h	38b (28.8)	94b (71.2)		
	>8 h	5b (29.4)	12b (70.6)		
Often in a state of high tension/stress	Often	4a (14.3)	24a (85.7)	5.47	0.01
	Occasionally	27a (23.3)	89a (76.7)		
	Never	12b (57.1)	9b (42.9)		
Consumption of yogurt, lactic acid bacteria beverages, and other probiotic products	Everyday	11a (35.5)	20a (64.5)	8.08	0.042
	Often	24a (30.8)	54a (69.2)		
	Occasionally	8b (14.3)	48b (85.7)		
Preference for acidic/sour flavor foods	No	25a (20.8)	95a (79.2)	11.73	0.012
	Yes	18b (40.0)	27b (60.0)		
Daily proportion of staple foods	Staple food as the main component	29a (20.9)	110a (79.1)	6.78	<0.01
	Staple food as the secondary component	14b (53.8)	12b (46.2)		

### 3.2 Analysis of gut microbiome diversity and composition

**Figure 1:**
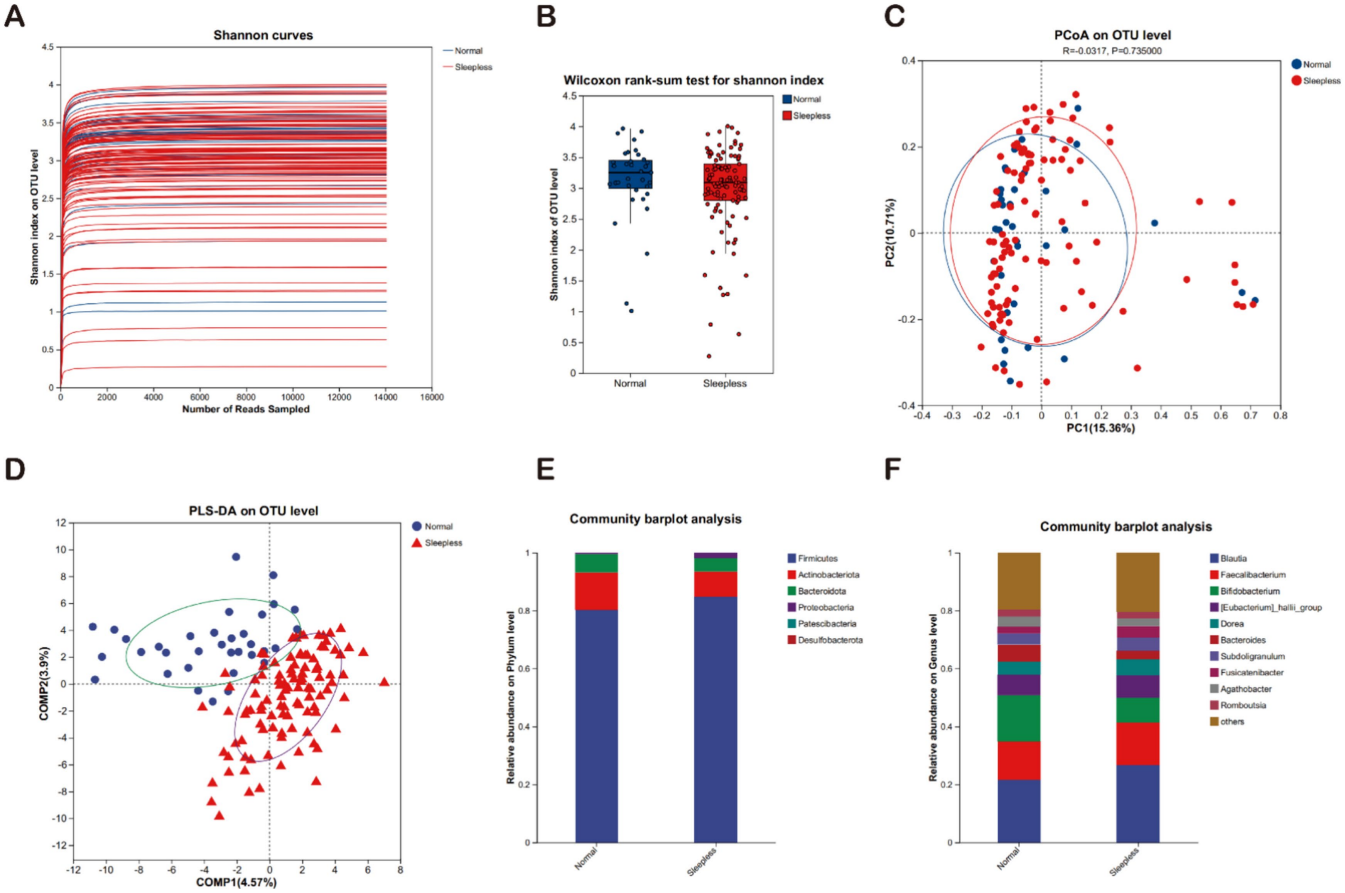
Fecal gut microbiome analysis. **(A)** Rarefaction curves of Shannon indices between the sleepless and normal groups. **(B)** Alpha diversity of Shannon indices between the sleepless and normal groups (^*^p > 0.05). **(C)** Beta diversity analyzed at the genus level using weighted UniFrac PCoA. **(D)** PLS-DA analysis of the microbial differences between the sleepless and normal groups. **(E)** Bar plot of the relative abundances of gut bacteria at the phylum level. **(F)** Bar plot of the relative abundances of gut bacteria at the genus level.

The rarefaction curves for the Shannon diversity index for each sample reached plateaus, indicating that the majority of the diversity was already procured (Figure 1A). The Shannon indexes (Figure 1B) used for alpha diversity analysis of metagenome data indicated no significant difference between the normal and sleepless groups, but we observed a tendency of decrease of the microbiome diversity in the sleepless. To evaluate the extent of the similarity of the bacterial communities, unweighted UniFrac principal component analysis (PCA) at the OTU level was employed and indicated no obvious separation between groups (Figure 1C). Then, partial least squares discriminant analysis (PLS-DA), a supervised analysis suitable for high-dimensional data, was performed (Figure 1D). The bacterial communities in the sleepless samples and the matched normal clustered separately, suggesting the overall structures of the bacterial communities in the groups were significantly different. These findings suggest that sleep deprivation may lead to substantial alterations in the composition of human gut microbiota.

The microbial communities were compared between the sleepless and control groups across different taxonomic levels. At the phylum level, the main phyla in both groups included Firmicutes, *Actinobacteriota*, and *Bacteroidota*. In the sleepless group, the proportions of Firmicutes and Proteobacteria phyla significantly increased, while the *Actinobacteriota* phylum significantly decreased (Figure 1E). At the genus level, the main genera in both groups included *Blautia*, *Faecalibacterium*, *Bifidobacterium*, *Eubacterium hallii group*, and others. In the sleepless group, the proportions of *Blautia* and *Eubacterium hallii group* genera significantly increased, while Bifidobacterium and Bacteroides genera exhibited the opposite trend (Figure 1F).

### 3.3 Analysis of gut microbiome differences

**Figure 2:**
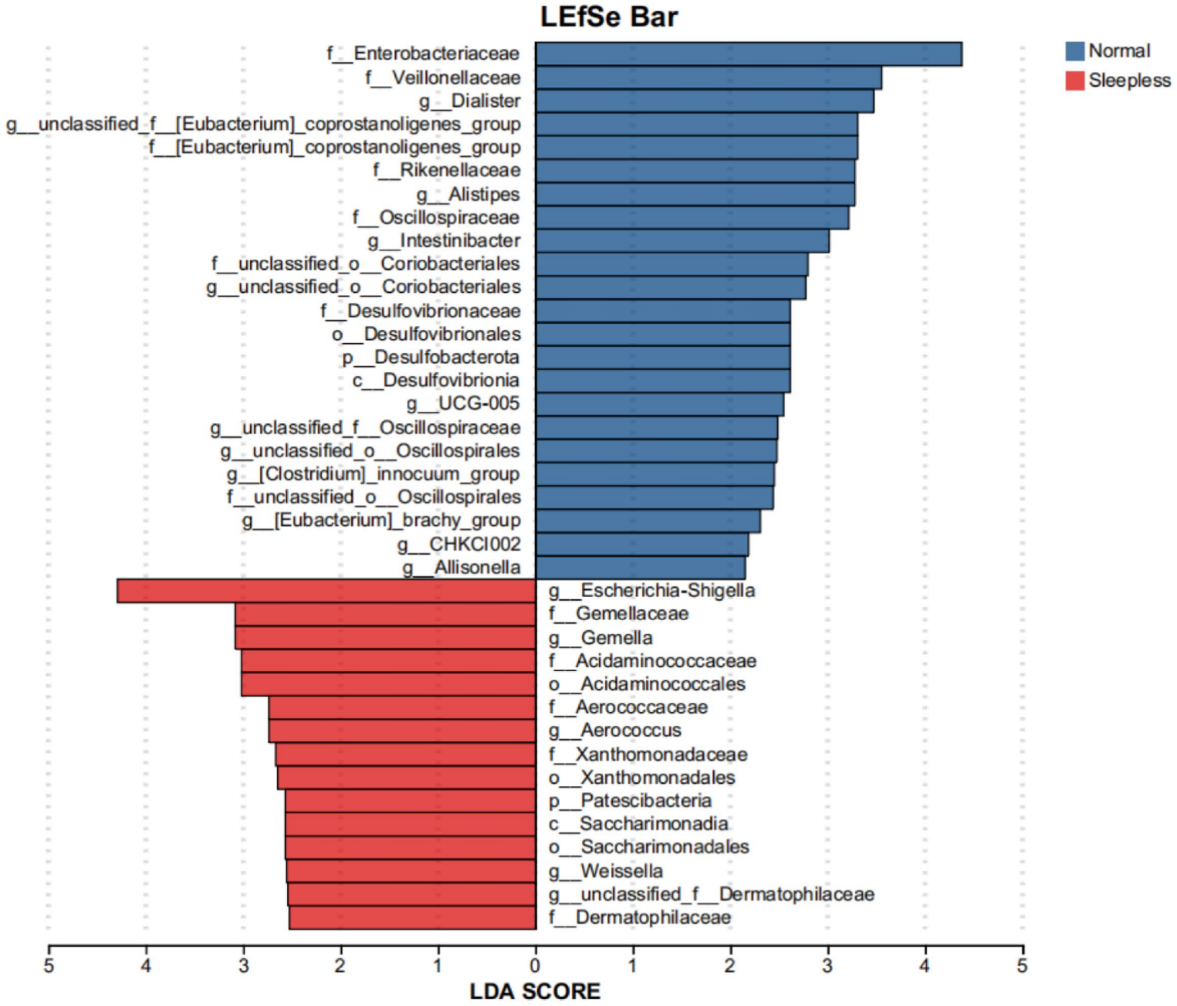
Bar plot of LEfSe analysis of the fecal gut microbiome. Orange and blue bars represent the degrees to which certain taxa are enriched in healthy controls and insomnia patients, respectively.

This study utilized LEfSe to compare the microbial community structures between the two groups. The results revealed 38 species with statistically significant differences (LDA scores >2), among which two species exhibited relatively larger differences (LDA scores >4). Specifically, in the sleepless group, the genus *Escherichia Shigella* was significantly enriched, while in the normal group, the family *Enterobacteriaceae* was significantly enriched. This indicates a compositional shift within Enterobacteriaceae under sleep deprivation, rather than a contradiction. These findings suggest that the gut microbiome composition of the sleepless group underwent significant changes, indicating a significant impact of sleep on the human gut microbiome (see Figure 2).

### 3.4 Analysis of gut microbiome metabolite characteristics

**Figure 3:**
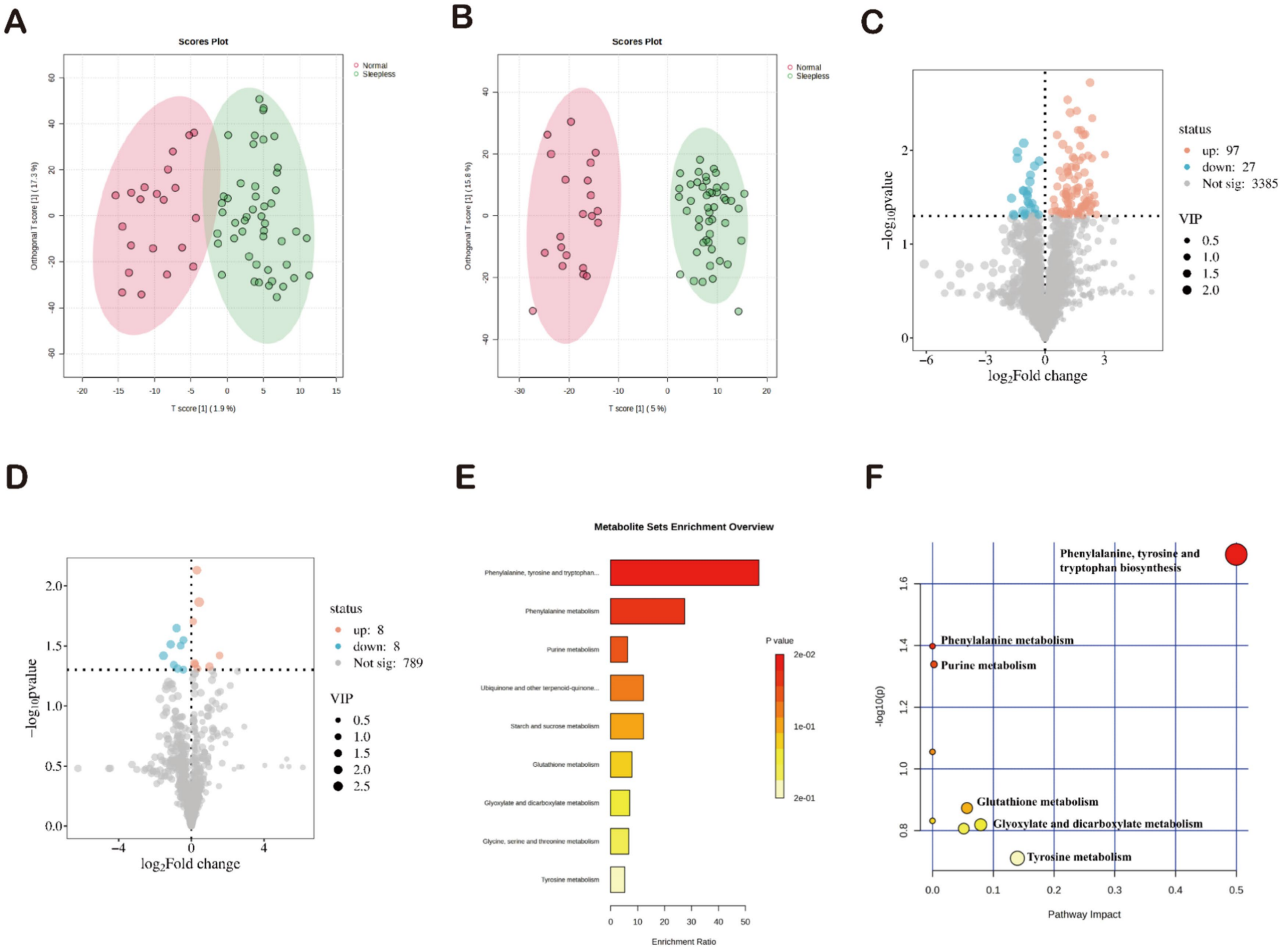
Characterization of gut microbiome metabolites in fecal samples. **(A,B)** Orthogonal partial least squares discriminant analysis of positive and negative ions. **(C,D)** Volcano plots of differential metabolites. **(E)** Enrichment analysis of 34 differential metabolites based on KEGG. **(F)** Pathway analysis of 34 differential metabolites based on KEGG.

Principal component analysis and orthogonal partial least squares discriminant analysis showed significant differences between the two groups, indicating significant differences in fecal metabolite composition between the normal and insomnia groups (Figures 3A,B). A total of 3,509 positive ion metabolites and 805 negative ion metabolites were identified in the two groups of samples. Using *p* < 0.05 and VIP ≥1 as criteria, 140 differential metabolites were screened in both ionic modes, with 97 increasing and 27 decreasing in positive ions (Figure 3C) and eight increasing and eight decreasing in positive ions (Figure 3D).

Subsequently, the identified differential metabolites underwent KEGG annotation, among which 34 differential metabolites were enriched. Functional pathway analysis of these differential metabolites using KEGG revealed their involvement in nine metabolic pathways. The three most significantly enriched pathways were “biosynthesis of phenylalanine, tyrosine, and tryptophan,” “phenylalanine metabolism,” and “purine metabolism” (*p* < 0.05) (Figure 3E). The pathway analysis results showed that the 34 differential metabolites were primarily involved in nine metabolic pathways. Consistent with the KEGG results, the three most significantly enriched pathways were “biosynthesis of phenylalanine, tyrosine, and tryptophan,” “phenylalanine metabolism,” and “purine metabolism” (*p* < 0.05) (Figure 3F). Therefore, we can infer that these three pathways may play a potential role in mediating the development of insomnia.

### 3.5 Analysis of oral microbiome composition and metabolite characteristics

**Figure 4:**
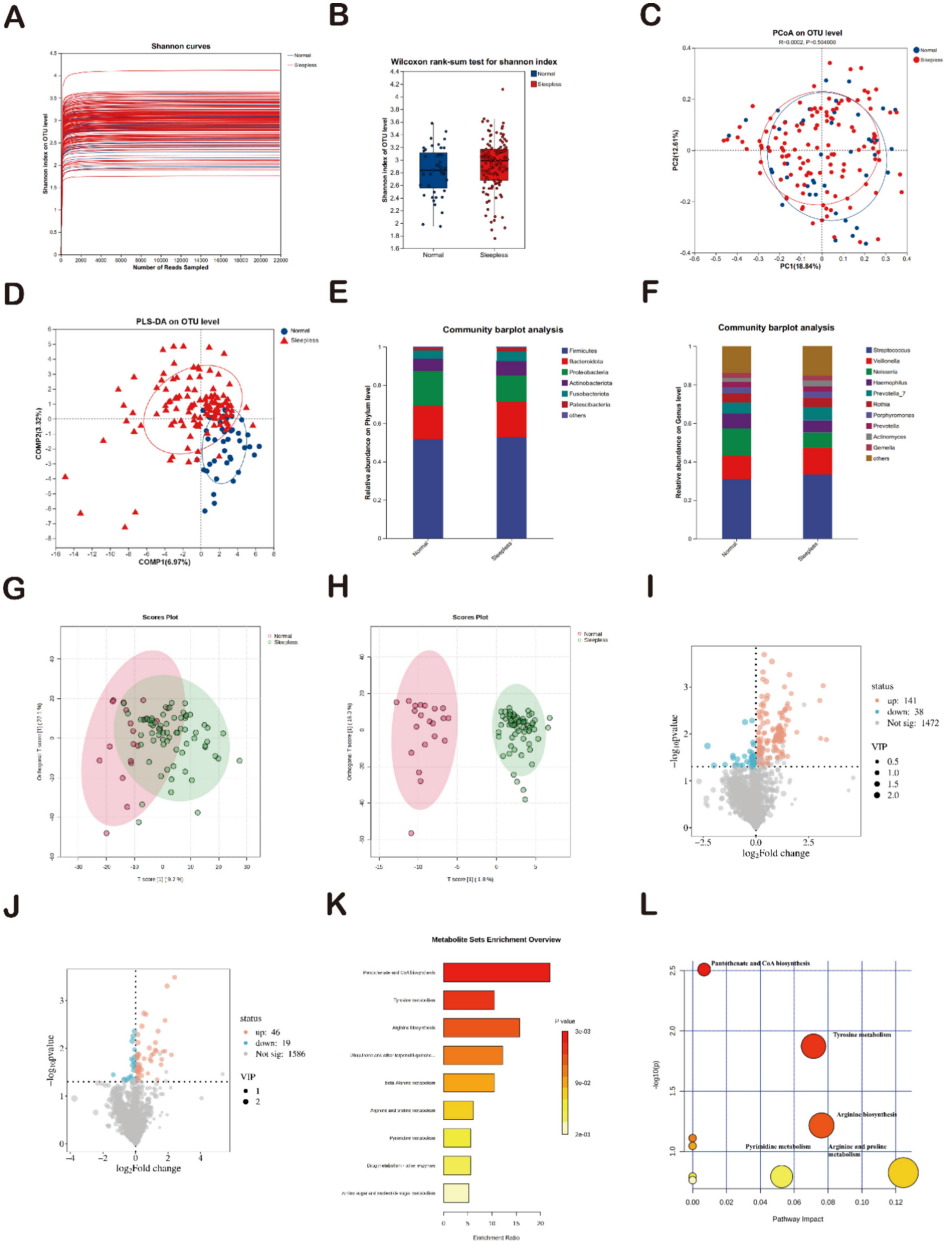
Analysis of salivary gut microbiome and metabolites. **(A)** Rarefaction curves of Shannon indices between the sleepless and normal groups. **(B)** Alpha diversity of Shannon indices between the sleepless and normal groups (^*^p > 0.05). **(C)** Beta diversity analyzed at the genus level using weighted UniFrac PCoA. **(D)** PLS-DA analysis of microbial differences between the sleepless and normal groups. **(E)** Bar plot of the relative abundances of gut bacteria at the phylum level. **(F)** Bar plot of the relative abundances of gut bacteria at the genus level. **(G,H)** Orthogonal partial least squares discriminant analysis of positive and negative ions. **(I,J)** Volcano plots of differential metabolites. **(K)** Enrichment analysis of 39 differential metabolites based on KEGG. **(L)** Pathway analysis of 39 differential metabolites based on KEGG.

The rarefaction curves for the Shannon diversity index for each sample reached plateaus, indicating that the majority of the diversity was already procured (Figure 4A). To elucidate the potential association between sleep and the gut-oral digestive microbiome, this study further analyzed the composition of the oral microbiome and its metabolite characteristics. Diversity analysis showed that the Shannon index exhibited an increasing trend in the sleepless group, which contrasted with the changes in gut microbiome diversity (Figure 4B). Regarding oral microbiome metabolite composition, weighted UniFrac PCoA analysis revealed no significant separation between groups (Figure 4C). Subsequently, PLS-DA analysis (Figure 4D) demonstrated distinct clustering of microbial communities in the sleepless and control samples, indicating significant differences in salivary metabolites between healthy individuals and those with insomnia. Spots representing samples from the sleepless group showed a more dispersed distribution pattern than those from the normal group, which is consistent with the increased level of bacterial diversity found in the insomnia samples. In terms of composition, the main phyla in both groups included Firmicutes, *Bacteroidota*, *Proteobacteria*, and *Actinobacteriota*. Compared to the gut microbiome composition, the proportion of Firmicutes decreased, while the proportions of *Bacteroidota* and *Proteobacteria* increased (Figure 4E). The main genera in both groups included Streptococcus, *Veillonella*, *Neisseria*, *Haemophilus*, and *Prevotella-7*. Compared to the gut microbiome composition, the proportions of Streptococcus, *Veillonella*, *Neisseria*, *Haemophilus*, and *Prevotella-7* genera were significantly higher (Figure 4F). A total of 1,651 positive ion metabolites and 1,651 negative ion metabolites were identified in the samples from both groups (Figures 4G,H). Using *p* < 0.05 and VIP ≥1 as criteria, 244 differential metabolites were screened in both ion modes (Figures 4I,J).

Subsequently, a total of 1,472 positive ion metabolites and 1,586 negative ion metabolites were identified in the two groups of samples. Using *p* < 0.05 and VIP ≥1 as criteria, 244 differential metabolites were screened in both ionic modes, with 141 increasing and 38 decreasing in positive ions (Figure 3C) and 46 increasing and 19 decreasing in positive ions (Figure 3D). Functional pathway analysis using KEGG revealed that the 88 differential metabolites were involved in nine metabolic pathways. The two most significantly enriched pathways were “biosynthesis of pantothenate and CoA” and “tyrosine metabolism” (*p* < 0.05) (Figure 4K). The pathway results showed that the 88 differential metabolites were mainly involved in nine metabolic pathways, with “biosynthesis of pantothenate and CoA” and “tyrosine metabolism” being the two most significantly enriched pathways (*p* < 0.05) (Figure 4L). This is highly consistent with the KEGG pathway analysis results. Therefore, we can infer that the two pathways “bio-synthesis of pantothenate and CoA” and “tyrosine metabolism” may play a potential role in mediating the development of insomnia. Compared to gut microbiome metabolites, “biosynthesis of pantothenate and CoA” was significantly enriched.

In summary, the composition of the oral microbiome and its metabolites in the poor sleep group exhibited significant changes compared to the normal group, and these changes were markedly different from the characteristics of the gut microbiome composition.

## 4 Discussion

Sleep quality is closely related to factors such as diet (Tanaka et al., 2023). For example, inadequate intake of vegetables and dairy products and excessive consumption of sweets increase the risk of insomnia (Arbués et al., 2019). Probiotics are an essential component of gut health, and their reduction may lead to gut dysbiosis, affecting the nervous system and sleep (Pala et al., 2024). This study suggests that moderate consumption of probiotic products is beneficial for sleep. Probiotics can improve sleep quality in patients with sleep disorders by restoring gut microbiome balance (Zeng et al., 2024).

Compared to the normal group, the gut microbiome diversity showed a decreasing trend in the sleepless group, which is consistent with previous findings that gut microbiome diversity is positively correlated with sleep efficiency and total sleep time (Smith et al., 2019). The absence of statistical significance observed in this study may be due to factors including inherent individual differences, the relatively small number of participants, or specific demographic characteristics of the selected population. Therefore, this finding should be interpreted with caution. Future investigations incorporating broader participant groups or more objective measurement techniques are needed to confirm and further explore this observed pattern. In this study, the dominant phyla in the sleepless and normal groups were *Firmicutes*, *Actinobacteriota*, and *Bacteroidetes*, indicating that the microbial compositions at higher taxonomic levels were similar between the two groups, representing the most important microbiome groups, which is consistent with previous reports (Olivia et al., 2020; Tong et al., 2022). At the genus level, the abundance of *Blautia* changed significantly, suggesting that insomnia may promote the proliferation of this genus, thereby affecting the microbiome structure (Migueis et al., 2019), further supporting the view that insomnia may disrupt the gut microbiome structure by affecting the relative abundances of specific genera (Migueis et al., 2019). Such alterations might result from mechanisms such as insomnia-induced stress hormones affecting gut motility (Konturek et al., 2011), immune system changes (Sun et al., 2023), or altered dietary behaviors (Sejbuk et al., 2024) associated with sleep disruption, ultimately influencing microbial proliferation and composition. LEfSe analysis found that *Escherichia-Shigella* was predominant in the sleepless group, and the compositions of gut metabolites differed significantly between the normal and sleepless groups (Zhou et al., 2022). KEGG functional pathway analysis of the differential gut microbiome metabolites revealed that the three most significantly enriched pathways were “biosynthesis of phenylalanine, tyrosine, and tryptophan,” “phenylalanine metabolism,” and “purine metabolism.” Among these, phenylalanine, tyrosine, and tryptophan are aromatic amino acids synthesized through the shikimate pathway and have important functions in biological systems. Phenylalanine can be converted to tyrosine, and the relationship between these metabolites and immune metabolism has been demonstrated in relevant studies (Geisler et al., 2013). Tryptophan is a precursor of 5-hydroxytryptamine (serotonin) and melatonin; high-carb diets promote Blautia-driven fermentation, increasing aromatic amino acid metabolites that alter serotonin synthesis, 5-hydroxytryptamine is involved in mood regulation, while melatonin plays a crucial role in regulating the sleep-wake cycle (Pandi-Perumal et al., 2007). Furthermore, abnormalities in the phenylalanine metabolism pathway are also associated with insomnia (Gordon-Dseagu et al., 2020). In addition, the enrichment of the “purine metabolism” pathway suggests potential links to sleep regulation, as purine metabolites like adenosine are known to play critical roles in promoting sleep drive and modulating arousal states, highlighting the need for further exploration of this metabolic signaling in the context of insomnia (Lazarus et al., 2019).

In the oral microbiome, the Shannon index curves of the sleepless and normal groups were overall similar, indicating no significant difference in species diversity between the two groups, which is consistent with the findings of Huang et al. (2024) but inconsistent with the findings of Liu et al. (2024); the slight increase in oral bacterial diversity in the sleepless group (*p* > 0.05) contrasts with gut trends and prior conflicting studies. This may be due to being influenced by stress-related dietary changes (e.g., high acid intake) or probiotic use, and further in-depth research is needed in the future. The main phyla in the sleepless and normal groups included *Firmicutes*, *Actinobacteriota*, *Bacteroidota*, and *Proteobacteria*, with overall similar distributions, suggesting that the insomnia state had little impact on the microbial composition at higher taxonomic levels. However, analysis at the genus level revealed significant abundance differences between the sleepless and normal groups. In the sleepless group, the relative abundance of *Streptococcus* decreased, while the abundance of *Veillonella* slightly increased, suggesting that the insomnia state may have influenced the oral microbiome. These changes can possibly come from stress, hormones, diet, or oral care interference. In the previous study, 85.7% of the sleepless group reported chronic high stress (vs. 14.3% in controls; *p* = 0.01), which may elevate cortisol and alter oral pH/mucosal immunity, favoring acid-tolerant Veillonella over Streptococcus. The sleepless group’s high acid food intake could suppress acid-sensitive Streptococcus. Sleep loss may reduce motivation for oral care, though we did not directly assess this.

This study found that the oral metabolite compositions differed between the normal and sleepless groups (Scholz et al., 2024). Insomnia is associated with changes in various metabolic pathways (Liang et al., 2022). KEGG annotation of the screened differential metabolites revealed that the two most significantly enriched pathways were “biosynthesis of pantothenate and CoA” and “tyrosine metabolism.” Acidic food preference linked to oral Veillonella, which thrives in low-pH environments and may disrupt oral-gut axis signaling. Veillonella utilizes lactate from acidic diets to produce propionate, which may inhibit pantothenate kinase, explaining the “pantothenate-CoA” pathway disturbance.

Pantothenate (vitamin B5) is a precursor for the biosynthesis of coenzyme A (CoA), which plays a key role in energy metabolism. Pantothenate is catalyzed by pantothenate kinase to form 4′-phosphopantothenate, which subsequently participates in the synthesis of CoA (Leonardi and Jackowski, 2007). CoA is essential in metabolic pathways such as fatty acid oxidation and the citric acid cycle (Xie et al., 2024). Pantothenate deficiency may lead to energy metabolism disorders, thereby affecting sleep quality (Leonardi and Jackowski, 2007). These results indicate that although the insomnia state did not significantly affect the overall species diversity of the oral microbiome, there were significant differences in the abundances at the genus level and the metabolite composition, suggesting that insomnia may further impact overall health status through metabolic dysregulation.

The microbiome-gut-brain axis (MGBA) closely links the gut microbiome with the nervous, endocrine, and immune systems, establishing a bidirectional regulatory pathway that is crucial for maintaining the dynamic balance of the sleep-wake cycle (Guo et al., 2024). This study speculates that the significant changes in the gut microbiome and metabolites may affect sleep quality through the MGBA by regulating neurotransmitters and immune functions. The combined therapy of oral and gut microbiomes is important for the recovery from various diseases (Shoer et al., 2023; Elghannam et al., 2024). However, our study has some limitations. First, the study population consisted of 165 adult males, and the results may not be generalizable to females or other age groups, and may need to be expanded to include more age groups. Secondly, there is the issue of sample collection. Saliva samples were collected “before brushing,” but oral hygiene habits (mouthwash use) were not controlled, which may have affected the composition of the flora. Thirdly, although we adjusted for key confounders, residual confounding from unmeasured dietary components (e.g., precise nutrient intake) cannot be excluded. Future studies should include detailed dietary records (e.g., 24-h recalls) to fully disentangle diet-sleep-microbiota interactions. Finally, the causality and mechanism of the study are not sufficiently explored to determine whether sleepless leads to changes in the flora, or flora disruption induces sleepless, the specific roles of salivary and fecal microbiomes in the pathogenesis of insomnia remain unclear, which can be further verified by fecal transplantation.

## 5 Conclusion

In summary, this study comprehensively compared the differences in gut and oral microbiomes, as well as metabolites, between the insomnia and healthy groups. Additionally, it identified microbial and metabolite biomarkers in saliva and feces for insomnia patients, providing new insights into the interactions among salivary and fecal microbiomes, immunity, and metabolites. Furthermore, it explored the mechanistic roles of salivary and fecal microbiomes and metabolites in insomnia, providing multi-omics data for further research on insomnia and laying an important foundation for subsequent large-sample validation and longitudinal studies.

## Data Availability

The datasets presented in this article are not readily available because of confidentiality regulations imposed by Institute of Food Science and Technology. Requests to access the datasets should be directed to the corresponding authors.
